# The cGAS‒STING pathway in colorectal cancer: bridging innate immunity and therapeutic strategies

**DOI:** 10.1186/s13046-025-03544-y

**Published:** 2025-10-08

**Authors:** Wen-Jing Li, Guang-Hui Dong, Yao Bi, You-Yan Han, Long-Long Sun, Tong Wang, Zhen-Hua Lin, Xiang-Shan Ren

**Affiliations:** 1 Central Laboratory, Yanbian University Hospital & Key Laboratory of Pathobiology, State Ethnic Affairs Commission, Yanbian University, Yanji, China; 2 Department of Anesthesiology, Affiliated Hospital of Yanbian University, Yanji, China; 3 Department of Neurology, Affiliated Hospital of Yanbian University, Yanji, China; 4 Department of Pathology & Key Laboratory of Pathobiology, Cancer Research Center, Yanbian University Medical College, State Ethnic Affairs, No. 977, Gongyuan Road, Yanji, 133002 China

**Keywords:** Colorectal cancer, Innate immunity, cGAS-STING pathway, Nanocarrier, Immunotherapy, Tumor microenvironment

## Abstract

Colorectal cancer (CRC) continues to be a predominant cause of cancer-related mortality worldwide, with existing therapies constrained by systemic toxicity, resistance, and inadequate tumor targeting. While immunotherapy has potential in specific CRC subtypes, its overall effectiveness is still limited. The cyclic GMP‒AMP synthase-stimulator of interferon genes (cGAS‒STING) pathway, an essential cytosolic DNA sensor that facilitates innate immune responses, has surfaced as a prospective target for cancer immunotherapy. Recent studies have demonstrated that it plays dual roles in CRC: on the one hand, it triggers antitumor immune responses, while on the other hand, it promotes intestinal inflammation. Accurate delivery of STING agonists made feasible by developments in nanotechnology offers novel ways to modify the TME and overcome resistance. The current understanding of the activation and function of the cGAS-STING pathway in CRC, its impact on the TME, and recent developments in STING-targeted therapeutic approaches, comprising monotherapy and combination strategies with chemotherapy, radiotherapy, and immune checkpoint inhibitors, is summarized in this review. We also review new nanomedicine approaches designed to increase STING activation. Understanding the complex roles of cGAS-STING in CRC could help guide the development of next-generation immunotherapies with improved selectivity and efficacy.

## Introduction and pathway basics

Colon cancer, or colorectal cancer (CRC), has become the most common malignancy affecting the gastrointestinal system worldwide. In the United States, CRC causes over 160,000 new cases and nearly 57,000 deaths each year, making it the second leading cause of cancer mortality [1]. CRC usually has no symptoms at early stages, resulting in late diagnosis and a high death rate [1]. The disease develops through genetic and epigenetic changes, typically progressing from harmless adenomatous polyps to invasive cancer [2, 3]. However, genetic changes alone do not cause CRC; a suppressive immune environment around the tumor and long-lasting inflammation also play important roles [4, 5]. For example, patients with long-term inflammatory bowel disease (IBD) have an increased risk of CRC due to constant inflammation in the colon. Recent studies have also shown that imbalances in the gut microbiota (dysbiosis) promote CRC by affecting inflammation and immunological reactions. This connection between genetics and the immune system supports the use of immunotherapy for CRC treatment [6, 7].

In the last ten years, immunotherapy has greatly improved cancer treatment [8]. Treatments such as immune checkpoint inhibitors, cancer vaccines, CAR-T-cell therapy, and adoptive T-cell therapy have been successful in treating many cancers [4, 5, 9–11]. However, immunotherapy works best in CRC cancers characterized by high microsatellite instability (MSI-H) or deficient mismatch repair (dMMR), which are more predisposed to respond to immunotherapy. Most CRC tumors are immunologically “cold” and do not respond well to immune checkpoint treatments [12, 13]. Therefore, there is great interest in finding ways to activate innate immunity and inflammation in tumors to improve immunotherapy. Therefore, the cyclic GMP–AMP synthase (cGAS)–stimulator of interferon genes (STING) pathway has become a promising target in cancer immunology.

The cGAS–STING pathway was first shown to defend against viruses by detecting cytosolic double-stranded DNA (dsDNA) [2, 5]. When unusual dsDNA from microbes or cells is found in the cytoplasm, an enzyme called cGAS produces a molecule called 2′,3′-cyclic GMP–AMP (cGAMP), which activates the protein STING. Activated STING triggers signals that produce type I interferons (IFN-Is) and proinflammatory cytokines, initiating a strong immune response [9, 14]. This pathway protects against infection but also affects cancer development and immune detection in tumors. Importantly, cGAS can detect abnormal DNA from tumor cells (such as micronuclei) and trigger immune responses against tumors through STING activation [15–18]. Because it activates immunity, the cGAS-STING pathway is a promising target for oncological therapy. STING agonists, including small molecules and cyclic dinucleotides (CDNs), strongly induce IFN-I and recruit immune cells, including antigen-presenting cells and cytotoxic T cells, to malignancies. This turns immunologically “cold” tumors into “hot” ones [19, 20]. Animal studies have shown that activating STING in tumors can stop tumor growth and increase the effectiveness of immune checkpoint treatments [21].

However, STING signaling must be carefully controlled, as this pathway can both fight tumors and promote inflammation-driven cancer. Short-term STING activation can stimulate antitumor immunity, but long-term STING activation can cause inflammation that promotes cancer growth [22]. In CRC, prolonged STING activation, such as in chronic inflammatory bowel disease, can increase inflammation and cancer risk, whereas brief and controlled STING activation can strengthen antitumor responses. Understanding the context-dependent effects of STING in CRC is crucial for safely targeting this pathway in therapy.

## Mechanisms of cGAS-STING activation/signaling

**Fig. 1:**
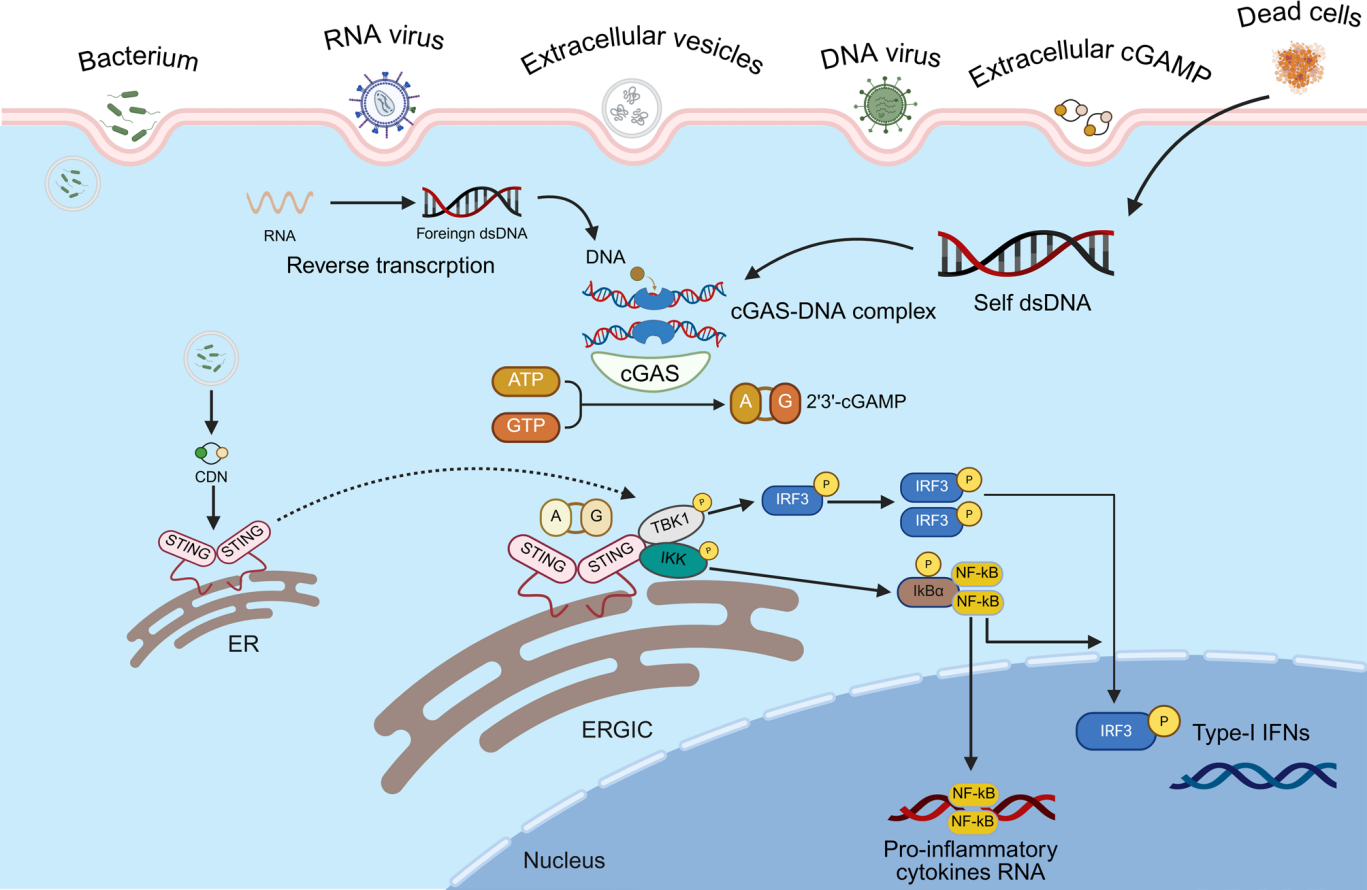
Molecular mechanisms underlying the cGAS-STING signaling pathway. Cytosolic dsDNA, originating from invading pathogens or endogenous sources such as damaged cells, is sensed by cGAS, which catalyzes the production of the second messenger cGAMP. cGAMP subsequently binds to STING, an adaptor protein located on the ER, triggering its activation and promoting its translocation to the Golgi apparatus via vesicular trafficking. In the Golgi apparatus, activated STING recruits and activates TBK1, which subsequently phosphorylates the transcription factor IRF3. Upon phosphorylation, IRF3 translocates to the nucleus, where it stimulates IFN-I expression. Simultaneously, STING signaling promotes the NF-κB pathway, resulting in the synthesis of proinflammatory cytokines. IFN-I and these cytokines collectively produce an innate immune response that facilitates the activation of adaptive immunity by enhancing the recruitment and stimulation of cytotoxic lymphocytes, such as CD8⁺ T cells and NK cells, thus contributing to antitumor immunological activity. Abbreviations: cGAS, cyclic GMP-AMP synthase; STING, stimulator of interferon genes; dsDNA, double-stranded DNA; cGAMP, cyclic GMP-AMP; ER, endoplasmic reticulum; TBK1, TANK-binding kinase 1; IRF3, interferon regulatory factor 3; IFN-I, type I interferon; NK, natural killer; NF-κB, nuclear factor-kappa B

To effectively target the cGAS-STING pathway in CRC therapy, it is critical to elucidate how this pathway is initiated and transmits signals. STING, an adaptor protein present in macrophages, dendritic cells(DCs), lymphocytes, and both endothelial and epithelial cells, was first discovered by Glen N. Barber’s group in 2008 [18]. This novel molecule exhibits pleiotropic properties within the immune system. STING serves as a critical activator in the sensing of pathogenic threats and initiates strong innate immune responses essential for host defense. Figure 1 depicts the cellular molecular mechanisms underlying cGAS-STING signaling. Under homeostatic conditions, STING resides on the endoplasmic reticulum (ER) membrane in an inactive state. The pathway is triggered when cGAS (located in the cytosol) binds to dsDNA that appears in the cytoplasm. Sources of such cytosolic DNA can include damaged host cell nuclei or mitochondria (self-DNA) as well as invading pathogens (foreign DNA from bacteria or viruses) [23–25]. Upon detecting DNA, cGAS experiences a conformational change that facilitates the production of the second messenger cGAMP, which utilizes ATP and GTP [26–28]. cGAMP subsequently binds to STING at the ER, promoting its dimerization and relocation to the Golgi apparatus [29]. STING recruits and activates TANK-binding kinase 1 (TBK1), which phosphorylates both STING and the transcription factor interferon regulatory factor 3 (IRF3) [30]. Phosphorylated IRF3 then dimerizes and moves into the nucleus, initiating the transcription of IFN-I genes and a wide spectrum of interferon-stimulated genes (ISGs) [31]. Simultaneously, the IκB kinase (IKK) complex is triggered by STING signaling, leading to the degradation of IκBα. IκBα is an inhibitor of nuclear factor kappa-B (NF-κB). This enables NF-κB to access the nucleus and stimulate the expression of proinflammatory cytokines, including interleukin-6 (IL-6) and tumor necrosis factor-alpha (TNF-α) [32]. The cGAS-STING pathway activates both the IRF3/IFN-I pathway and the NF-κB pathway, thereby orchestrating a potent innate immune response and supporting adaptive immunity by enhancing antigen presentation and T-cell activation.

Notably, the effects of the cGAS-STING pathway vary on the basis of the situation. Temporary stimulation induces protective antitumor immunity, whereas chronic overactivation can lead to pathological inflammation [33]. For example, persistent STING signaling is linked to conditions such as colitis, which can promote CRC over time. Cells have evolved negative feedback mechanisms to restrain excessive STING activation [34]. A considerable number of CRC cells exhibit impaired cGAS‒STING signaling. Certain tumors exhibit mutations that cause decreased functioning or reduced expression of cGAS or STING, a phenomenon that may facilitate their evasion of immune surveillance [35]. One study revealed that STING expression was undetectable in 36% of human colon cancers, and such loss of STING correlated with an impaired DNA damage response and accelerated tumor growth [36]. Conversely, restoring STING signaling in those cells could reinstate immune surveillance. These findings underscore that whether tumor cells possess an intact cGAS‒STING pathway profoundly affects CRC development and the effectiveness of treatment.

Additionally, the DNA damage response of tumors forms a key link between cGAS-STING signaling and CRC outcomes. Mechanistic data, in addition to correlative results, show that genomic instability directly triggers the STING pathway. In CRC cells, DNA damage induced by radiation or chemical agents can activate the cGAS–STING pathway, resulting in the production of IFN-I, which facilitates the recruitment of DCs and T cells [37, 38]. Importantly, whether a tumor cell has a functional cGAS–STING pathway plays a key role in determining how sensitive it is to treatment and how strongly the immune system is activated.

Overall, the cGAS-STING pathway functions as a significant detector of the cell’s cytosolic DNA, which in turn activates the production of interferons as well as various cytokines in response to perceived threats to the genome. In CRC and other cancers, its activation can help initiate immune recognition by tumor cells. The following sections explore its distinct roles in intestinal inflammation and cancer and evaluate strategies to leverage this pathway for therapeutic purposes.

## Context-dependent effects (tumor suppressive vs. promoting)

**Fig. 2:**
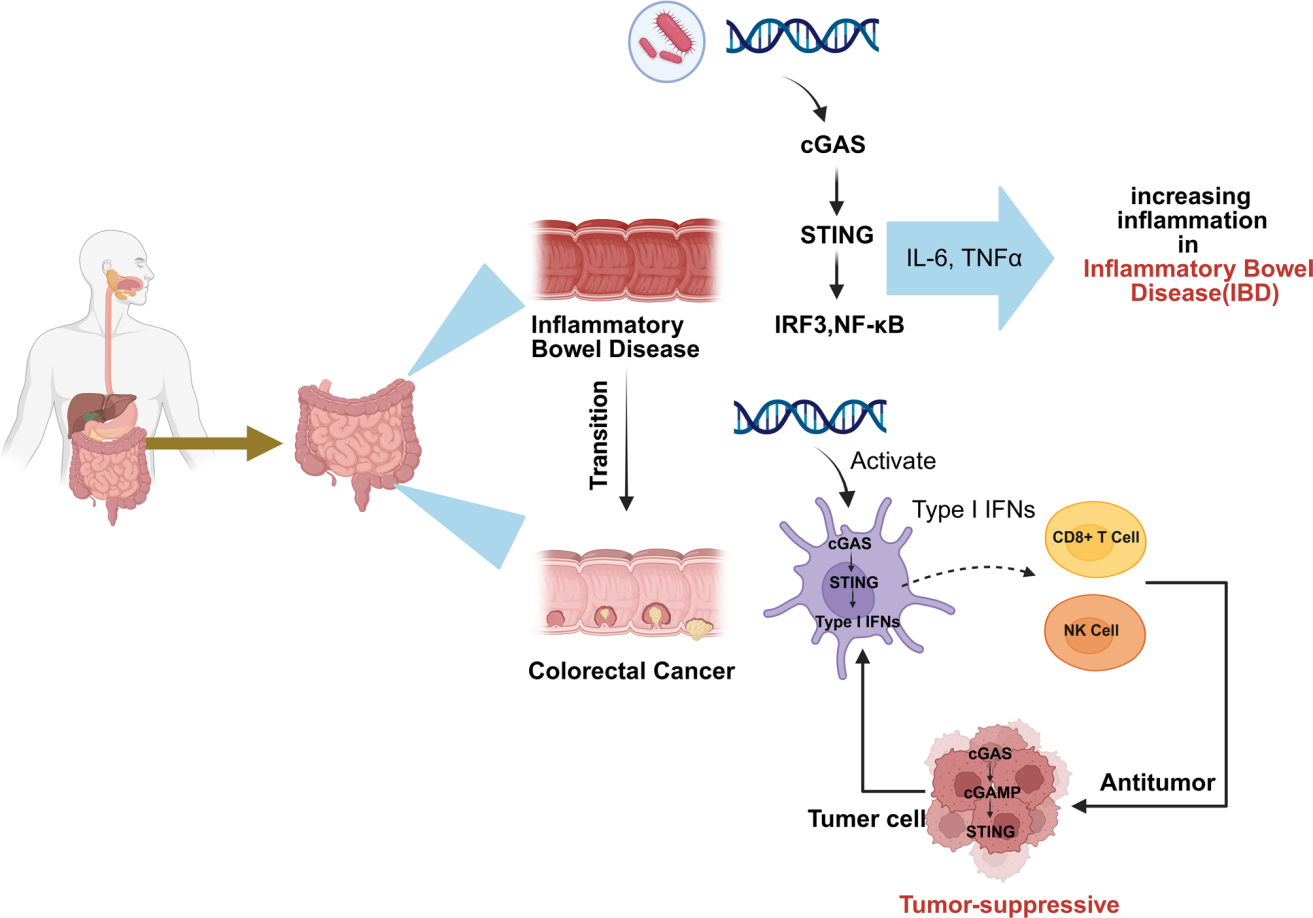
The dual functions of the cGAS-STING pathway in IBD vs. CRC. In IBD, microbial and host-derived DNA stimulate the cGAS‒STING pathway, leading to IRF3-mediated production of proinflammatory cytokines such as IL-6 and TNF-α, which exacerbates intestinal inflammation. In contrast, in CRC, the activation of an identical signaling cascade stimulates IFN-I production and promotes the recruitment of CD8⁺ T cells and NK cells, thereby augmenting antitumor immune responses

In colorectal disease, the cGAS‒STING pathway senses DNA in cells and has two different roles. It can cause inflammation and activate antitumor immunity [39]. Researchers have shown that cGAS-STING signaling has dual effects on tumor progression that depend on context [39]. In chronic inflammatory conditions (such as IBD), continuous activation of cGAS-STING creates a tumor-promoting environment [40]. In established CRC, short-term STING activation usually improves antitumor immunity (Fig. 2). In addition to activating tumor immunity, the dual effects of the cGAS‒STING pathway are also manifested in its direct cytotoxic effects on tumor cells. The following section will further elaborate on the dual roles of the cGAS‒STING pathway.

### Tumor-promoting inflammatory functions of cGAS-STING in IBD-associated CRC

The cGAS-STING signaling pathway plays a multifaceted role in chronic intestinal inflammatory conditions, including IBD, which encompasses Crohn’s disease and ulcerative colitis [41]. During the transition from IBD to CRC, inflammation that promotes tumors is caused by hyperactivation of the cGAS-STING pathway. Increasing evidence suggests that persistent STING signaling fosters chronic, unresolved inflammation, thereby facilitating tumor initiation and progression. Chronic inflammation in IBD can aberrantly activate cGAS-STING, exacerbating tissue damage [42, 43]. STING works by turning on the NF-κB and IRF3 pathways, which in turn increases the transcription of IFN-I and inflammatory cytokines [39, 44]. These inflammatory mediators create a positive feedback loop by bringing in and activating more immune cells. They can also act on epithelial cells to cause more stress and DNA damage, which keeps the inflammation going in the mucosa [45]. For instance, the activation of STING in lamina propria macrophages and DCs significantly affects the immune microenvironment in individuals with colitis by altering macrophage polarization and cytokine release [40].

Importantly, abnormal STING signaling in the intestinal epithelium itself also plays a role in tumor growth caused by inflammation. Intestinal epithelial cells (IECs) under stress or genomic injury can activate cGAS-STING upon exposure to cytosolic DNA, resulting in autocrine and paracrine inflammatory signaling. Recent studies have shown that a loss of genomic stability in IECs can trigger inflammation mediated by cGAS-STING. For instance, the removal of the helicase DExH-box Helicase 9 (Dhx9) in the mouse intestinal epithelium leads to the buildup of RNA hybrids and cytosolic DNA, which triggers strong STING-dependent interferon responses [46]. The result is serious damage to the epithelium: mice lacking Dhx9 have problems with renewing intestinal stem cells, damage to the barrier, and worse colitis, which can be improved by blocking STING with drugs [46]. These findings suggest that excessive STING activation serves as a central driver of inflammation-associated epithelial impairment and intestinal tissue injury, thereby promoting conditions conducive to dysplasia and tumor development. Clinical samples from ulcerative colitis patients exhibit upregulated STING pathway signaling, and immune cells have been identified in both IECs and the lamina propria, correlating with active disease. In colonic organoid cultures, inflammatory cytokines linked to IBD, including interferon beta (IFN-β) and TNF-α, were demonstrated to synergistically hyperactivate STING signaling, resulting in enhanced inflammasome activation and epithelial cell death [47–49]. These STING-driven cycles of epithelial injury and mortality, succeeded by regenerative proliferation, can foster an environment conducive to the accumulation of oncogenic mutations and facilitate the progression from chronic colitis to CRC.

In vivo IBD/CRC models also support the idea that STING overactivation causes tumors to grow. It has been shown that pharmacological hyperactivation of STING can make colitis worse. In a murine model, the administration of the STING agonist vadimezan (DMXAA) significantly aggravated dextran sulfate sodium (DSS)-induced colitis, resulting in a substantial decrease in body weight [50]. This heightened inflammation is recognized to facilitate tumorigenesis by causing DNA damage and establishing an immunosuppressive, wound-healing tissue milieu. On the other hand, removing STING from genes can temporarily reduce damage caused by inflammation. For example, STINGknockout mice have less severe acute colitis, which shows how STING drives inflammation in the mucosa [50]. However, when it comes to the long-term growth of cancer, not having STING can actually make tumors bigger, which shows how this pathway can work in two ways. Gong W et al. [51] showed that Tmem173 (STING knockout mice) had less severe colitis but more tumors related to colitis than wild-type mice. These animals with low levels of STING have CRC that grows faster, along with an inflammatory tumor microenvironment (TME) that favors protumor immune cell populations and uncontrolled epithelial growth [51]. On the other hand, mice with STING showed fewer tumors, which suggeststhat STING signaling starts inflammation that leads to tumors but also starts immune responses that stop tumors from growing. In fact, studies have shown that activating STING can control the intrinsic properties of tumor cells (like growth, adhesion, and invasion) as well as the functions of immune cells that fight cancer in the CRC setting [52]. The net effect of chronic STING signaling in IBD is therefore a double-edged sword, but evidence clearly indicates that uncontrolled STING driven inflammation strongly favors tumorigenesis in the early stages [39].

### Tumor-suppressive functions of cGAS-STING in CRC

#### STING-mediated programmed cell death pathways

**Fig. 3:**
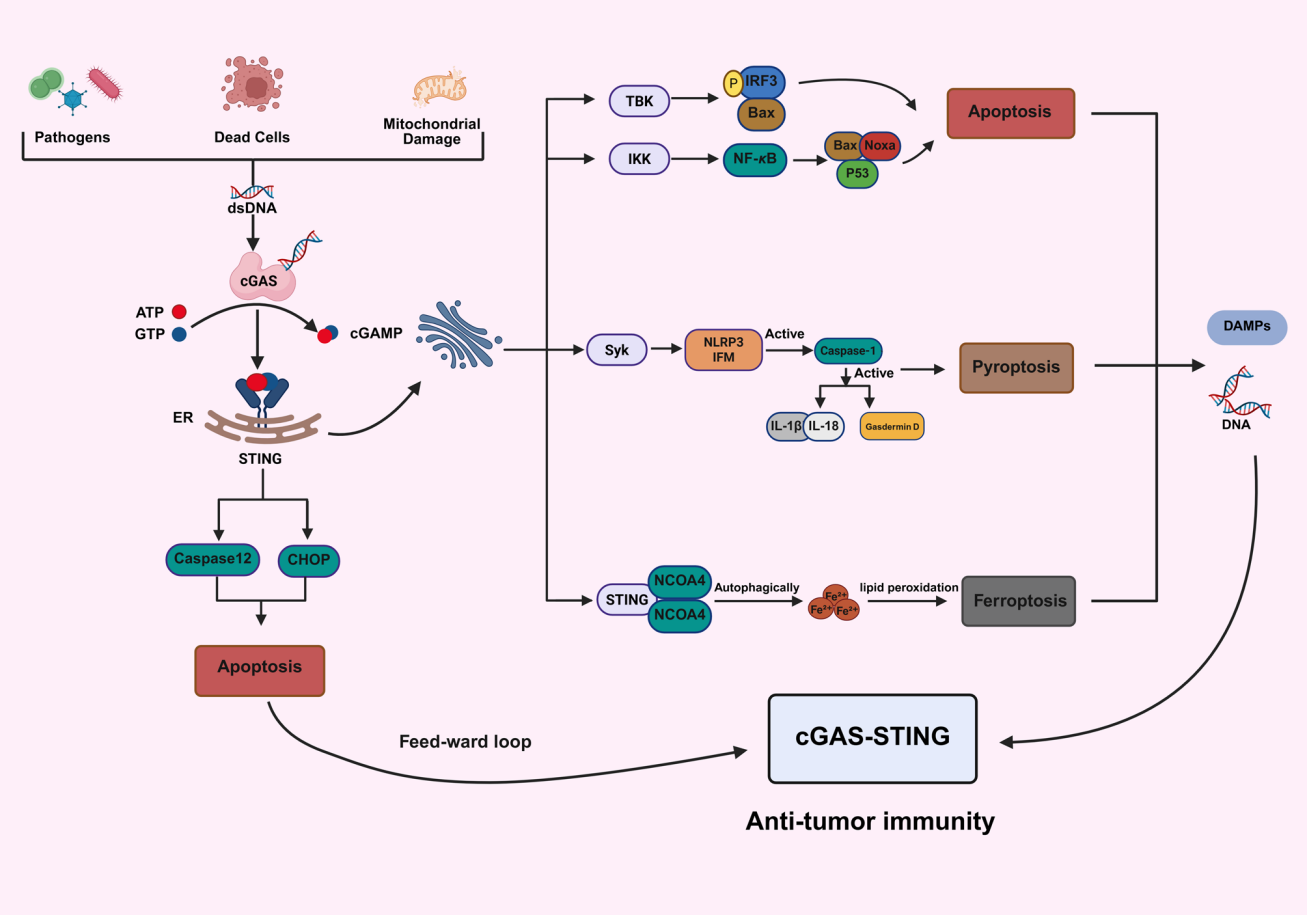
cGAS-STING as a Hub Linking Innate Sensing and Tumor Cell Death in CRC. Cytosolic dsDNA in CRC cells is sensed by cGAS, which catalyzes the synthesis of 2′3′-cGAMP; cGAMP then binds to STING on the endoplasmic reticulum. Activated STING engages multiple cell death and stress pathways: it drives apoptotic signaling via IRF3- and NF‑κB-dependent transcription and induces an unfolded protein/ER stress response (involving the IRE1/CHOP and PERK pathways, with caspase‑12 activation). In parallel, STING recruits Syk kinase to trigger the NLRP3 inflammasome, causing caspase-1-mediated gasdermin D cleavage and the release of IL-1β/IL-18 (pyroptosis). STING also binds the ferritinophagy receptor NCOA4, initiating ferritin degradation and the release of Fe²⁺, which drives lipid peroxidation-dependent ferroptosis. All of these death pathways liberate danger signals (e.g., DAMPs and DNA) that are captured by dendritic/immune cells and sensed via cGAS-STING (activating TBK1-IRF3 and IFN-I), creating a feedforward loop that amplifies type-I interferon-mediated antitumor immunity

In CRC, cGAS–STING signaling is predominantly regarded as protective, exerting tumor-suppressive effects via both direct tumor cell killing and the activation of antitumor immunity [53]. The essential intracellular mechanism entails the commencement of programmed cell death (Fig. 3). STING activation has been demonstrated to elicit apoptosis, pyroptosis, or ferroptosis [54]. In CRC models, STING engages each of these proteins: for example, IRF3 downstream of STING upregulates proapoptotic BH3-only proteins (Noxa/PUMA) to activate Bax-dependent mitochondrial apoptosis [55]; STING can also prime NF-κB/NOD-, LRR-, and pyrin domain-containing protein 3 (NLRP3) to cleave caspase-1 and gasdermin D (GSDMD) for pyroptosis [56]; and STING intersects ferroptosis regulators such as nuclear receptor coactivator 4 (NCOA4), a key regulator of ferritinophagy and the antioxidant glutathione peroxidase 4 (GPX4)–solute carrier family 7 member 11 (SLC7A11) axis to control lipid peroxidation [57, 58]. These forms of cell death not only eliminate tumor cells directly but can also release tumor antigens and danger signals that further stimulate immune responses.

However, Among the connections is death. Via downstream effectors (e.g., NF-κB, IRF3, or ER stress pathways), the cGAS-STING pathway can initiate apoptotic cell death [59]. Reciprocally, cytosolic DNA released by dead cells may activate cGAS-STING [60], establishing a feedback loop between cell death and the immune response. cGAS–STING activation can promote intrinsic apoptosis in CRC. For example, STING-driven IRF3 phosphorylation induces transcription of the p53-responsive proapoptotic proteins Noxa and Puma, which activate Bax and mitochondrial cytochrome c release [55]. Conversely, the executioner caspase-3 provides negative feedback: active caspases cleave cGAS and IRF3, thereby dampening the DNA-sensing response [61]. B-cell lymphoma 2 (BCL-2) also links these pathways. In CRC cells, BCL-2 binds to mitochondrial voltage-dependent anion channel protein 1 (VDAC1) and prevents mtDNA leakage and STING activation. If BCL-2 is blocked (for example, with ABT-199), this stops the interaction between BCL-2 and VDAC1. These results reveal how Bax, BCL-2, and caspase-3 are connected to cGAS-STING signaling in colorectal tumors [62]. Although more research on STING-induced death in CRC is needed, it is likely involved in immune-mediated tumor cell clearance [63]. One fascinating finding is that cGAS-STING activation in the TME can stimulate immunogenic cell death, a type of tumor cell death or necrosis that releases tumor antigens and adjuvant signals, thus enhancing immunity [64].

Beyond apoptosis, STING signaling is also implicated in pyroptosis and necroptosis, two lytic cell death pathways that can shape tumor inflammation [65]. Pyroptosis is a type of caspase-1-mediated inflammatory cell death that is typically triggered by inflammasome activation. STING has been demonstrated to activate the NLRP3 inflammasome under specific conditions [66]. In CRC cells, forced NLRP3 activation was found to increase STING-dependent IFN-β and C-X-C motif chemokine ligand 10 (CXCL10) production and CD8^+^ T-cell immunity [67]. Interestingly, this effect occurred even when caspase-1 was inhibited. Gong et al. [51] indicated that in colitis-associated cancer models, STING engaged with spleen tyrosine kinase (Syk) and facilitated NLRP3 inflammasome activation, resulting in heightened epithelial cell pyroptosis and inflammation. Chronic STING-driven pyroptosis in normal epithelial cells is bad, but acute, tumor-specific pyroptosis through STING activation could help get rid of cancer cells and release new antigens. On the other hand, it might be good to control pyroptosis in tumors. A recent study found that activating STING along with some chemotherapeutics caused gasdermin-mediated pyroptosis in CRC cells. This boosted the immune system and slowed the growth of the tumor [68].

Among the various forms of cell death associated with cGAS-STING signaling, ferroptosis—an iron-dependent programmed necrosis marked by lipid peroxidation—has recently attracted considerable attention. Ferroptosis is becoming a possible treatment target for CRC [69]. Crosstalk occurs between ferroptosis and STING signaling: cells undergoing ferroptosis can release damage-associated molecular patterns (DAMPs) that activate STING, which may subsequently influence their sensitivity to ferroptosis [70]. Recent research revealed a vital component of STING in regulating ferroptosis via direct engagement with NCOA4, thus revealing a link between innate immune sensing and iron homeostasis. The activation of the cGAS-STING pathway facilitates NCOA4-mediated ferritinophagy, a process involving ferritin degradation through the process of autophagy. This process releases redox-active iron, which fuels Fenton chemistry and causes lethal lipid peroxidation [71, 72]. These findings suggest that STING activation can be harnessed to induce ferroptosis in tumors [72]. Mechanistically, cyclic-dinucleotide interactions occur between the binding domain of STING and the coiled-coil domain of NCOA4, a physical association that initiates ferritinophagy-dependent ferroptosis and concurrently stabilizes STING dimers, thereby enhancing downstream inflammatory signaling [71]. Consistently, inhibiting STING or chelating iron blunts NCOA4-driven ferritinophagy and protects against ferroptotic damage in injury models [73]. In solid tumors, especially colorectal and pancreatic cancers, this STING-NCOA4 axis has dual-edged implications. On the one hand, STING-induced ferritinophagy can promote tumor cell ferroptosis and the release of DAMPs, which may augment antitumor immunity by recruiting and activating cytotoxic T cells [74–76]. STING pathway activation is widely recognized to induce IFN-I and proinflammatory cytokines, fostering T-cell infiltration in otherwise “cold” TMEs (as observed in STING agonist-treated pancreatic tumors) [74]. On the other hand, ferroptotic cell death can provoke inflammatory feedback; for example, in hepatocellular carcinoma models, ferroptotic tumor cells activate STING in macrophages, leading to interleukin 1 beta (IL-1β) release and a protumorigenic immunosuppressive milieu [77, 78]. These findings underscore that the STING-NCOA4 interaction serves as a critical nexus between ferritinophagy and immune signaling in ferroptosis. Therapeutically, targeting this interplay is promising—for example, combining STING agonists with ferroptosis inducers has been demonstrated to elicit robust immunogenic cell death in CRC models—highlighting a novel strategy to kill tumor cells while simultaneously engaging the immune system [78, 79].

On the other hand, high expression of GPX4 (and SLC7A11) protects CRC cells. GPX4 overexpression is linked to poor patient survival [57, 58]. Blocking GPX4 can also improve immunotherapy. For example, the GPX4 inhibitor RSL3 greatly improved anti–programmed cell death protein 1 (PD-1) effects by causing lipid peroxidation in CRC models [80]. In addition, new agents (such as N6F11 or vitamin C plus cetuximab) cause GPX4 degradation through the proteasome. This causes ferroptosis and HMGB1 release, which then activates CD8^+^ T cells [81]. Similarly, reducing SLC7A11 (the cystine transporter) causes ferroptosis. For example, the natural product ginsenoside Rh3 lowers SLC7A11 through the signal transducer and activator of transcription 3 (STAT3) / tumor protein p53 (p53) / nuclear factor erythroid 2–related factor 2 (NRF2) pathway, reduces glutathione (GSH) levels, and enhances immune checkpoint inhibitor (ICI) responses in CRC [82]. Another study revealed that interferon output downstream of STING activation may alter the cellular redox state or modulate immune cell recruitment in a manner that enhances ferroptosis within the TME [83]. In CRC, ferroptosis is associated with autophagy-dependent mechanisms and has been demonstrated to enhance antitumor immunity via the release of immunogenic signals [84]. A promising treatment technique involves the concurrent production of ferroptosis within neoplastic cells and STING activation in immune cells, which is an approach that has a synergistic effect by directly eradicating cancer cells while simultaneously alerting and activating the immune system. An example of this is the outer membrane vesicle (OMV)/SaFe nanoparticle platform created by Sun et al. [85]. They electrostatically anchored iron ions to OMVs, functionalized them, loaded them with STING agonist-4, modified them with tumor-targeted 1,2-distearoyl-sn-glycero-3-phosphoethanolamine-N-[folate(polyethylene glycol)] (DSPE-PEG-FA), and engineered bacterial outer membrane vesicles loaded with iron Fe^2+^ to induce ferroptosis in colon cancer cells. These OMVs (called “SaFeFAs”) cause extensive lipid peroxidation and tumor cell death and stimulate DCs via STING activation (likely through tumor cell DNA or mitochondrial DNA released during ferroptosis). In colon cancer mouse models, OMV/SaFeFA therapy significantly inhibited tumor proliferation and improved survival, with evidence of robust CD8⁺ T-cell activation. These results suggest that ferroptosis-induced cell death can work with STING-mediated immune activation to create a strong antitumor effect. In a similar vein, Zhang et al. [86] demonstrated that the combination of a ferroptosis inducer with a STING agonist resulted in “triple-pathway” activation (STING, ferroptosis, and other forms of immunogenic cell death), yielding enduring antitumor immunity and immune memory in CRC models.

Necroptosis, a form of caspase-independent necrotic cell death, is part of another feedback loop. Necroptosis in tumors can liberate cytosolic DNA from dying cells, consequently activating cGAS-STING within the local milieu [87]. For instance, when tumor cells experience necroptosis as a result of chemotherapy or radiation, the released DNA can activate STING in host immune cells, thereby enhancing antitumor immunity [88]. However, too much necroptosis can also make the microenvironment immunosuppressive if it causes long-term inflammation. Careful calibration is needed to harness cell death–STING interactions for therapy.

#### STING-driven activation of antitumor immunity

In addition to these direct anti-tumor mechanisms, during colorectal tumorigenesis, accumulated DNA damage and genomic instability generate cytosolic DNA in cancer cells (e.g., from micronuclei formed due to chromosome missegregation) [33]. This activates cGAS-STING within tumor cells and surrounding immune cells, leading to interferon production and the recruitment of immune effectors that can attack tumors by Li A et al. [89] Indeed, multiple studies have correlated activated STING signaling with better clinical outcomes in patients with CRC. Yang et al. [90] found that patients with CRC who exhibited elevated intratumoral STING expression experienced increased CD8^+^ T-cell penetration, reduced rates of metastasis, and considerably improved overall and relapse-free survival compared with those with low STING activity. A study used mice to look into how injecting the STING agonist 3’3’-cGAMP directly into colon tumors affects their growth. The study’s results showed that this type of injection greatly slowed the growth of colon tumors by encouraging CD8^+^ T-cell infiltration and activation [91]. These results suggest that STING activity in the TME creates an environment rich in cytotoxic T cells that slows the growth of CRC. In terms of how it works, STING can be turned on in many different types of cells in the TME. For example, tumor cells that still have STING can make IFN-I when their DNA is damaged, and immune cells in the host (like DCs) can sense DNA or cGAMP from the tumor and start an interferon response [21]. STING-driven IFN-I improves the cross-priming of CD8⁺ T cells and the maturation of DCs, linking innate sensing to adaptive immunity against tumors [20, 92]. On the other hand, if the STING pathway is suppressed or nonfunctional in a tumor, the cancer can more readily evade immune detection. For example, cGAS or STING gene silencing in CRC cells can lead to decreased chemokine production and reduced T-cell recruitment [34]. Restoring STING in such tumors (or delivering a STING agonist to the TME) can reverse this immune evasion strategy by triggering local innate immunity [93].

The cGAS-STING signaling pathway demonstrates tumor-suppressive effects in CRC via two interconnected mechanisms: the induction of programmed cell death in malignant cells and the initiation of robust anti-tumor immune responses. This process results in the direct elimination of tumor cells and promotes the release of tumor antigens and danger signals, enhancing immune recognition and response. The STING pathway facilitates the production of interferon and cytokines, which in turn recruit and activate DCs and cytotoxic T lymphocytes, thereby connecting innate DNA sensing with adaptive tumor-specific immunity. A feedback loop is present between cell death and immune activation; for example, cytoplasmic DNA released from dying tumor cells can activate the cGAS-STING pathway in the TME, thereby enhancing immune surveillance. The consequences of STING signaling are context-dependent. Activation of STING, specific to tumors, induces immunogenic cell death and robust antitumor immune responses. Conversely, chronic or dysregulated STING activity, frequently linked to ongoing inflammation, can establish an immunosuppressive inflammatory microenvironment that paradoxically facilitates tumor growth. Next section will examine the context-dependent interaction between STING-mediated cell death and immunoregulation, with a focus on the various immunological functions of STING in the TME.

### The immunomodulating roles of STING in the CRC TME

**Fig. 4:**
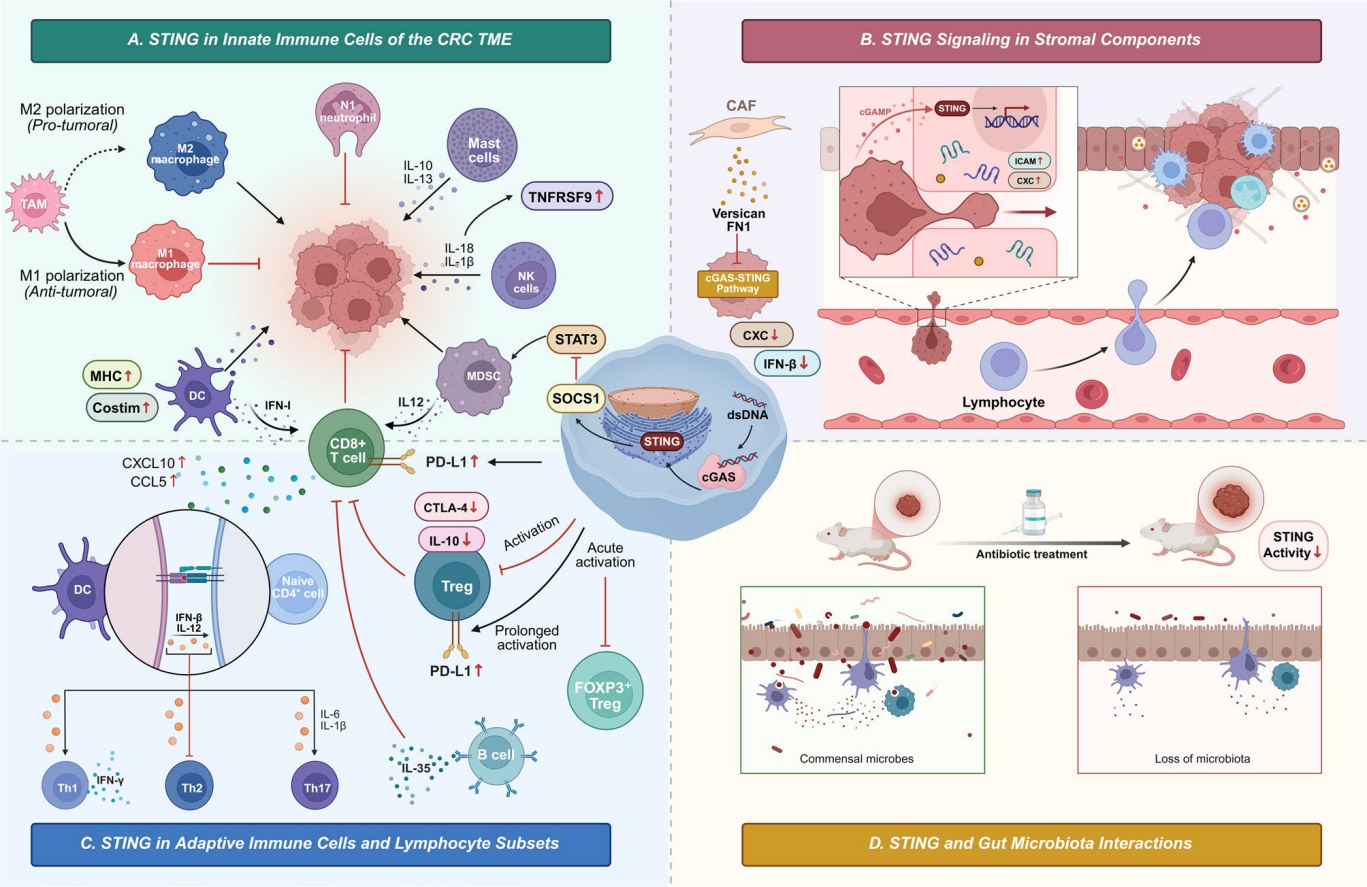
Multifaceted roles of STING signaling in the CRC TME. (**A**) STING in innate immune cells of the CRC TME: STING promotes M1 macrophage polarization and suppresses M2-like tumor-associated macrophages (TAMs); it activates dendritic cells (DCs), NK cells, and N1 neutrophils; modulates myeloid-derived suppressor cells (MDSCs) and mast cells via the SOCS1/STAT3 pathway; and enhances CD8⁺ T-cell function. (**B**) STING signaling in stromal components: in endothelial cells, STING increases immune infiltration via ICAM and chemokines; cancer-associated fibroblasts (CAFs) suppress STING signaling via versican and fibronectin-1 (FN1), reducing IFN-β production. (**C**) STING in adaptive immune cells and lymphocyte subsets: STING supports Th1 differentiation, modulates regulatory T cells (Tregs) and B cells, and promotes type I interferon (IFN-I) production and T-cell priming. (**D**) STING and gut microbiota interactions: Commensal microbes sustain STING activity, whereas antibiotic treatment or microbiota loss reduces STING signaling and impairs immune surveillance

Building on the above mechanisms, the immunomodulatory impact of STING in the CRC TME is highly context-dependent. STING signaling increases DC activation, T-cell infiltration, and antitumor immunity in CRC [94]. Nevertheless, the TME is immunosuppressive and marked by an abundance of M2-polarized tumor-associated macrophages (TAMs), regulatory T cells (Tregs), and myeloid-derived suppressor cells (MDSCs) and increased levels of cytokines, including TNF-α, IL-1β, and IL-6 [95, 96], with microbiota dysbiosis further impairing immune activation [97]. Figure 4 illustrates STING signaling across the immune, stromal, and microbial compartments of the CRC TME, highlighting its integrative role in coordinating tumor–immune–microbial interactions.

#### STING in innate immune cells in the CRC TME

STING in the CRC TME influences innate immunity by modulating macrophages, DCs, natural killer (NK) cells, neutrophils, and mast cells. This modulation regulates cytotoxicity, antigen presentation, and inflammatory tone, ultimately affecting tumor development and therapeutic response. TAMs in the CRC microenvironment can polarize into either the immunosuppressive M2 phenotype or the proinflammatory M1 phenotype within these innate compartments. STING signaling increases antitumor immunity by enhancing M1 polarization and suppressing M2 properties [98]. Driving metabolic reprogramming in TAMs [99] induces immune-responsive gene 1 (IRG1). Under hypoxia, tumor-derived exosomal microribonucleic acids (microRNAs) suppress STING, thus reducing the expression of IFN-I and C-X-C motif chemokine ligand 9 (CXCL9) [100, 101]. These results emphasize that STING is a fundamental control of the macrophage phenotype and immune modulation in colon cancer.

Neutrophils and MDSCs: Neutrophils in tumors can similarly adopt antitumor (N1) or protumor (N2) functional states. MDSCs, which are often similar to neutrophils in CRC, are important for immunosuppression [102, 103]. STING signaling can deactivate suppressive neutrophils/MDSCs and encourage their transformation into immunostimulatory cells. STING agonists stimulate MDSCs to secrete interleukin-12 (IL-12) and other proinflammatory mediators, thereby augmenting CD8^+^ T-cell responses [104]. STING activates the suppressor of cytokine signaling 1 gene (SOCS1), which stops the activation of STAT3. This makes MDSC-derived immunosuppressive factors less effective and changes these cells into a more antitumor phenotype [98]. Conversely, context is significant: Therapy-induced DNA damage can activate STING in tumor cells, which can increase the levels of chemokines that bring neutrophils and MDSCs to tumors. This could create an environment that suppresses the immune system [105]. Consequently, STING exerts dual effects on neutrophils. The intrinsic activation of STING within cells generally enhances N1-like antitumor functions, whereas STING-mediated signals originating from tumor cells may recruit granulocytic cells that inhibit T-cell activity.

DCs: When cGAS-STING is turned on, DCs make IFN-I and increase the levels of major histocompatibility complex (MHC) and costimulatory molecules [106]. In CRC, STING activation in DCs encourages the self-presentation of tumor antigens, stimulates CD8⁺ T-cell activation [107], and facilitates the recruitment of basic leucine zipper ATF-like transcription factor 3 (Batf3)⁺ DCs and cytotoxic lymphocytes. STING agonists enhance DC-mediated T-cell priming and synergize with checkpoint inhibitors [97, 108]. Low STING expression is correlated with poor DC maturation and a non-inflamed TME. STING thus bridges innate DNA sensing and adaptive T helper type 1 (Th1)-polarized antitumor immunity in CRC.

NK cells: The prompt doesn’t specifically mention NK cells, but they are important innate lymphocytes in CRC whose activity can be changed by STING through macrophage cross-talk. Recent research has shown that STING-activated macrophages can enhance the cytotoxicity of NK cells. In a model of CRC liver metastasis, the activation of STING in macrophages triggered NLRP3 inflammasome signaling, resulting in the synthesis of interleukin-18 (IL-18) and IL-1β. These cytokines subsequently upregulate the costimulatory receptor tumor necrosis factor receptor superfamily member 9 (TNFRSF9) on NK cells and TNFSF9 on macrophages, thereby markedly enhancing NK cell-mediated antitumor activity [109]. The absence of STING in myeloid cells hindered NK cell activation and facilitated metastatic proliferation. So, even though NK cells have low levels of STING, the STING pathway in TAMs can boost NK cell-mediated immunity through signals that depend on cytokines and cell contact [109]. These results show how STING controls the cooperation of innate immune cells to stop CRC from spreading.

Mast cells: Tumor-infiltrating mast cells are associated with angiogenesis and immunosuppression in CRC [110]. Direct data on STING in mast cells are limited. However, STING-induced IFN-Is might affect mast cell activity. IFN-Is can stabilize the vasculature and modulate mast cell degranulation, potentially counteracting the protumor effects of mast cells. Conversely, mast cells release cytokines, which include interleukin 10 (IL-10) and interleukin-13 (IL-13), that can suppress STING-driven inflammation. Further research is needed, but it is conceivable that STING agonism within the TME might restrain mast cell-mediated tumor promotion by shifting the cytokine milieu toward a Th1/IFN-dominated state.

#### STING signaling in stromal components

STING also acts on stromal cells in CRC. In endothelial cells, STING enhances immune cell trafficking and vessel normalization, whereas in cancer-associated fibroblasts (CAFs), suppressed STING activity contributes to immune exclusion and tumor evasion.

Endothelial cells: The tumor endothelium acts as both a barrier and a gateway for immune infiltration. STING activation can prompt endothelial cells to respond, leading to vascular normalization and increased lymphocyte trafficking. A recent study using an orthotopic model demonstrated that cGAMP produced by tumor cells can travel through gap junctions involving leucine-rich repeats containing 8 VRAC subunit C (LRRC8C) channels to activate STING in endothelial cells [111]. STING activation upregulated adhesion molecules and chemokines, facilitating T-cell adhesion and transendothelial migration into tumors [112]. Moreover, STING-driven signaling reduces abnormal angiogenesis and tumor hypoxia, creating a more immune-permissive microenvironment [113]. These results reveal a novel form of intercellular communication in which tumor-intrinsic DNA sensing induces STING signaling in host endothelial cells, resulting in vascular remodeling that facilitates immune cell infiltration and strengthens antitumor immunity in CRC. Therapies that stimulate STING, such as STING agonists.

NPs have demonstrated the ability to decrease tumor vessel density and improve perfusion and T-cell entry [113]. Thus, the function of STING signaling in stromal endothelial cells is crucial for regulating both the structural and immunological elements of the TME because of its action on immune cells.

CAFs: CAFs are key constituents of the stromal microenvironment in CRC, influencing immune responses through the secretion of soluble factors and extracellular matrix remodeling. Recent studies have shown that high CAF infiltration, as indicated by versican expression, is associated with decreased STING expression in tumor cells [114]. In vitro, primary CAFs suppress cGAS and STING in CRC cells, likely through factors such as versican and fibronectin-1. This downregulation diminishes IFN-β and chemokine production, which impairs T-cell recruitment and promotes immune evasion. While inflammatory fibroblast subsets can support STING under certain conditions, most tumor CAFs contribute to the immune-excluded TME. Restoring STING signaling by targeting CAF-tumor interactions may increase the efficacy of immunotherapy [115, 116].

#### STING in adaptive immune cells and lymphocyte subsets

CD8⁺ Cytotoxic T lymphocytes: STING signaling facilitates the onset of adaptive antitumor immunity by augmenting CD8^+^ T-cell responses. The introduction of IFN-I enhances dendritic cell activation and aids in the cross-priming of tumor-specific cytotoxic T lymphocytes. STING also increases the levels of chemokines like CXCL10 and chemokine ligand 5 (CCL5), which helps CD8^+^ T-cells get into colorectal tumors [117, 118]. Elevated STING expression is associated with Th1-polarized responses and a favorable prognosis in CRC [91]. STING agonists make CD8⁺ T cells more active and able to get into tumors, which makes tumors more sensitive to PD-1 or programmed cell death ligand 1 (PD-L1) immune checkpoint blockade [119]. However, prolonged STING activation due to persistent DNA damage can enhance the expression of immunosuppressive factors like PD-L1 and indoleamine 2,3-dioxygenase (IDO), potentially compromising T-cell functionality [120]. In summary, the regulated activation of the STING pathway has been shown to enhance CD8^+^ T-cell-mediated antitumor responses in CRC.

CD4^+^ helper T cells: Cytokines from activated DCs and macrophages affect CD4^+^ T-cell differentiation indirectly through STING. IFN-β and IL-12 help Th1 cells become polarized and make IFN-γ while stopping T helper 2 (Th2) cells from becoming skewed. The absence of STING is associated with elevated expression of GATA-binding protein 3 (GATA3), signifying a transition towards Th2 or regulatory phenotypes [119, 121]. IL-1β and IL-6 that are made by STING may help Th17 cells develop, but Th17 cells can also do two things at once in CRC. Chronic STING signaling, exemplified by tumor-derived micronuclei, can activate NF-κB and STAT3, promoting protumor Th17 responses [122, 123]. So, STING usually helps Th1 immunity, but when it is out of control, it may help tumors grow by Th2 or Th17.

Tregs: STING has context-dependent effects on Tregs. Acute activation reduces Forkhead box protein P3 (FOXP3)⁺ Tregs in CRC, which is increasing effector T-cell activity [124]. STING-induced IFN-I downregulates Treg-recruiting chemokines and impairs Treg function. STING activation within Tregs inhibits the expression of suppressive genes, which include IL-10 and cytotoxic T-lymphocyte-associated protein 4 (CTLA-4), and it promotes CD4^+^ T-cell proliferation [125, 126]. In contrast, prolonged or tumor-intrinsic STING activity may increase Treg abundance via feedback. IFN-β can drive IL-10 production by myeloid cells, and that also can enhance Treg induction and PD-L1 expression [125, 127]. Additionally, tumor-derived exosomes may activate STING in naïve CD4⁺ T cells, leading to FOXP3⁺ Treg conversion [128].

B cells: B cells in CRC can function as antigen-presenting cells and antibody producers but may also acquire regulatory phenotypes. STING activation has been shown to induce interleukin-35 (IL-35)-producing B cells that suppress NK and CD8⁺ T-cell activity [97]. In addition to its role in CRC, this immunosuppressive mechanism may also apply to CRC. Conversely, STING activation is correlated with B-cell recruitment and tertiary lymphoid structure formation, which are linked to better outcomes. In intestinal immunity, STING-deficient mice demonstrate diminished immunoglobulin A (IgA) production attributable to microbial dysbiosis [129]. In general, STING may improve the immunostimulatory or regulatory functions of B cells, depending on the situation. However, its role in CRC is still not fully understood.

#### Interactions between STING and the gut microbiota

Emerging evidence indicates that the gut microbiota serves as a key external modulator of STING signaling in CRC. The CRC patients exhibit microbial dysbiosis with reduced intratumoral STING-related protein expression. In mouse models, antibiotic-induced microbiota depletion expedites tumor proliferation and inhibits STING activation [130]. These results indicate that commensal microbes maintain basal STING activity, which also enhances antitumor immunity. The loss of the microbiota interrupts this pathway, making it easier for the immune system to avoid detection. The microbiome, consequently, impacts the tumor immune microenvironment by regulating STING-mediated surveillance and may influence cancer progression and therapeutic response [130].

In conclusion, STING acts as a double-edged sword in the CRC TME, capable of inducing robust antitumor immunity while, under specific circumstances, facilitating protumor immunosuppression. On one hand, activating the STING pathway can change myeloid cells into a proinflammatory, tumoricidal type, improve the function of dendritic cells and T-cell priming, increase the number of cytotoxic lymphocytes that enter the tumor, and even make the tumor’s blood vessels more normal. All of these changes work together to slow tumor growth and make the tumor more responsive to immunotherapy. Conversely, persistent or contextually inappropriate STING signaling may promote tumor progression by perpetuating chronic inflammation and creating an immunosuppressive environment. Extended STING activity can increase the expression of inhibitory molecules like PD-L1 and IDO, while promoting the accumulation of MDSCs and Tregs, thus hindering effective T-cell responses. The overall effect of STING in the CRC TME is contingent upon the interplay between its immunostimulatory and immunosuppressive effects. Comprehending this duality is crucial for clinical implementation. This supports the therapeutic targeting of the cGAS–STING pathway, enhancing STING’s tumor-fighting capacity while mitigating its tumor-promoting tendencies. Next section will analyze innovative therapeutic approaches to modulate STING signaling in CRC, with the objective of utilizing its antitumor effects to improve patient outcomes.

## Therapeutic targeting strategies

STING pathway activation in CRC drives a potent innate-adaptive immune cascade. Activated STING induces robust type I IFN production and DC cross-priming, which causes expansion and tumor infiltration of CD8^+^ T cells. In addition, it triggers tumor/stromal secretion of chemokines (such as CXCL9/10 and CCL5), which recruit NK and effector T cells into the tumor [91]. Moreover, IFN-driven STING signaling feeds back to increase the number of immune checkpoints.

### Novel STING agonists

Because the cGAS-STING pathway can induce both innate and adaptive immune responses, it is becoming an increasingly attractive target for cancer immunotherapy. Nontucleotide small-molecule compounds as well as CDNs that resemble the natural ligand cGAMP have been developed as STING agonists. Among these agents, several are under clinical review in solid tumors [131]. These agonists seek to induce an in situ vaccination effect by turning the tumor into a source of immunogenic antigens and thus fostering systemic antitumor immunity by activating STING within the TME. In combination with other therapeutic modalities [45, 132, 133], in preclinical CRC models, STING agonists have been shown to be rather successful in reducing tumor growth. Nevertheless, species-specific variations in the STING structure present a translational challenge. For example, the small molecule DMXAA is inactive in human cells but powerfully activates murine STING [134]. To address this, next-generation STING agonists—such as [P(R)]-5′-O-[(R)-hydroxymercaptophosphinyl]-P-thioadenylyl-(2′→5′)-adenosine cyclic nucleotide and disodium salt (ADU-S100)—have been engineered for selective binding to human STING; as of now, no STING agonist has been approved clinically, but several are in phase I‒II trials. In early-phase trials, intratumoral administration of ADU-S100 in patients with advanced solid neoplasms, including metastatic colorectal carcinoma, induced local immune activation, although monotherapy responses were limited [135]. Current clinical strategies are exploring their utilization alongside immune checkpoint inhibitors to augment therapeutic efficacy [19].

### Synergy with checkpoint blockade

While STING agonists can enhance immune cell infiltration in immunologically “cold” colorectal tumors, their effectiveness as monotherapies is frequently limited by the intricate immunosuppressive milieu of the TME. A more effective approach involves combining STING pathway activation with immune checkpoint inhibitors. In a murine CRC model, the noncyclic dinucleotide (non-CDN) STING agonist diamidobenzimidazole STING agonist-1 (diABZI), when combined with an IDO inhibitor that disrupts immunosuppressive tryptophan metabolism and anti-PD-1 therapy, significantly enhanced tumor suppression relative to monotherapy with any single agent [136]. In terms of how it works, STING activation increases immune infiltration, IDO inhibition reduces local T-cell suppression, and PD-1 blockade keeps T-cell effector function going. This multimodal strategy successfully transforms otherwise unresponsive tumors into immune-sensitive lesions [136].

Encouraging outcomes have also been reported when STING agonists are combined with immune checkpoint inhibitors for the treatment of MSI-H CRC [124]. Because MSI-H tumors already exhibit partial activation of cGAS-STING signaling, it is hypothesized that pharmacological STING stimulation could further amplify antigen presentation and immune priming, potentially broadening the subset of patients responsive to PD-1 blockade. Notably, increased cGAS expression in MSI-H CRC has been suggested as a potential predictive biomarker for enhanced responses to immune checkpoint therapy [124]. STING agonists may also sensitize “checkpoint nonresponders” by sufficiently inflaming the TME to enable T-cell infiltration and activation. Ongoing clinical investigations are evaluating the effectiveness of STING agonists combined with anti-PD-1 therapy in gastrointestinal cancers, including CRC, to assess their synergistic potential.

On the other hand, some evidence indicates that standard chemotherapy might have unintended consequences via STING. Research conducted by Liang et al. [137] emphasizing the “antagonistic” dual function of 5-FU + oxaliplatin (FOLFOX) chemotherapy in CRC, which is mediated by STING signaling. However, FOLFOX chemotherapy can exert dual effects via STING signaling: DNA damage from FOLFOX activates STING and upregulates IFN-β (which is useful for the immune system against tumors), but this same STING activation induces robust expression of PD-L1 on neoplastic cells, possibly helping them escape immune surveillance [137]. In CRC models, blocking the PD-1/PD-L1 pathway improved the effectiveness of FOLFOX, suggesting that chemoinduced STING activation should be paired with checkpoint inhibition to unleash full antitumor potential. The implication is that while STING stimulates immunity, tumors may counteract this effect by increasing the expression of immune checkpoints, a tug-of-war that combination therapy can resolve.

### Combining STING agonists with chemo- and radiotherapy

Traditional treatments such as chemotherapy and radiotherapy induce immunogenic DNA damage, providing a mechanistic rationale for combination with STING agonists. Systemic administration of STING agonists during cytotoxic treatment may enhance immune surveillance by priming the host immune system to recognize antigens from dying tumor cells. For example, oxaliplatin is a standard DNA-damaging agent in CRC that can induce immunogenic cell death that can be potentiated by concurrent STING activation [21]. Similarly, ionizing radiation increases the amount of cytosolic DNA in both the tumor and stromal compartments, initiating the cGAS-STING pathway.

One way to achieve this goal involves targeting DNA repair pathways, for example, the inhibition of Rad3-related (ATR) (a vital DNA damage checkpoint kinase), which results in STING activation and immune cell infiltration in CRC models in combination with radiation [13]. Liu et al. [13] found that an ATR inhibitor plus radiotherapy led to increased STING activation and immune cell infiltration in colorectal tumors, partly by suppressing Src homology region 2 domain-containing phosphatase-1 (SHP1). This strategy turned on antitumor immunity and improved tumor control. Similarly, chemotherapeutic DNA-damaging agents can inadvertently trigger STING [138]. 5-Fluorouracil (5-FU), a backbone chemotherapeutic for treating CRC, was recently found to require tumor cell-intrinsic STING activity for full efficacy [137]. Tian and colleagues revealed that 5-FU-mediated DNA damage in CRC cells activates the cGAS‒STING pathway and induces IFN-I production, which recruits immune cells that clear tumor cells [139]. 5-FU has lost most of its antitumor effect on STING-deficient tumors [139]. Thus, immunomodulation, which depends on functional STING signaling inside the TME, mediates some 5-FU antitumor effects. These observations suggest that combining genotoxic treatments with interventions to sustain or increase STING signaling could cooperatively improve antitumor immunity.

Another relevant molecule is SHP2, a phosphatase involved in oncogenic signaling. Wei et al. [140] discovered that activating the phosphatase SHP2 (for example, with the agonist lovastatin) impaired poly [ADP‒ribose] polymerase 1 (PARP1)-mediated DNA repair. This led to accumulation of cytosolic DNA, heightened STING activation, and increased IFN production, ultimately sensitizing tumors to chemotherapy. These findings pave the way for the development of adjuvant therapies that deliberately augment cGAS-STING signaling within neoplastic cells as a means to improve the efficiency of conventional treatments.

**Fig. 5:**
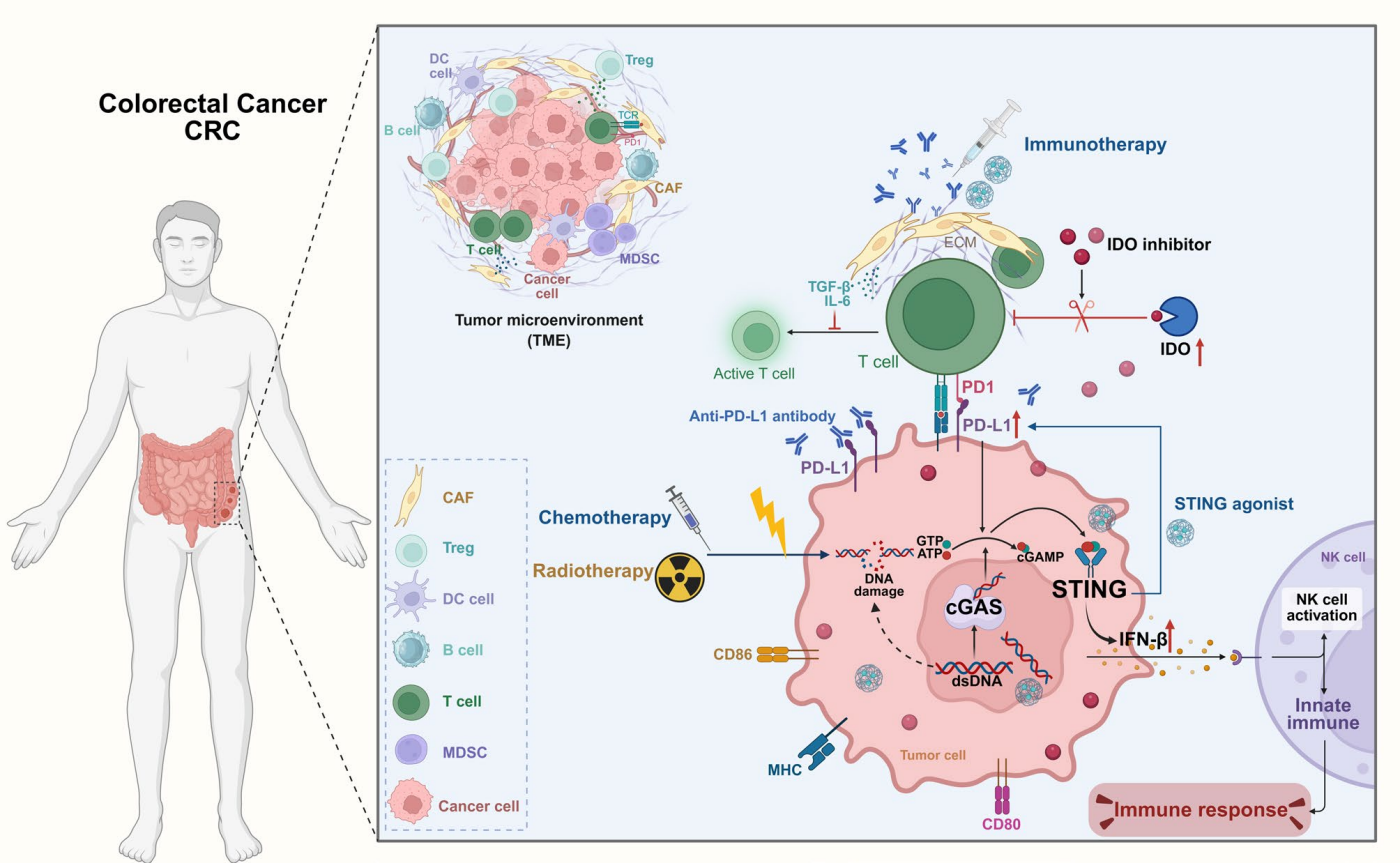
TME in CRC and therapeutic modulation of the cGAS–STING pathway. The left panel shows CRC localization and key TME components (cancer cells, CAFs, Tregs, DCs, B cells, and MDSCs). The right panel shows the ability of cGAS to sense cytosolic dsDNA and synthesize cGAMP from ATP/GTP. cGAMP binds STING, activating TBK1/IRF3 to induce type I IFNs (e.g., IFN-β) and immune responses. STING agonists or DNA-damaging therapies (chemotherapy/radiotherapy) further amplify this signaling pathway. STING activation promotes antigen presentation (upregulation of CD80/CD86 on DCs), NK cell activation, and PD-L1 expression on tumor cells. Immune checkpoint blockade agents (anti-PD-L1 antibodies) and IDO inhibitors synergize with STING activation to overcome TME immunosuppression

Preclinical investigations have demonstrated that the combination of radiotherapy and STING agonists can elicit abscopal effects, with regression of nonirradiated distant tumors mediated by IFN-I-driven systemic T-cell responses [141]. This rationale is being tested in the STING agonists formulated into cancer vaccines (STINGVAX) trial, which combines localized radiotherapy and intratumoral ADU-S100 injection in CRC liver metastases, aiming to achieve systemic tumor control. Figure 5 provides a visual summary of STING pathway activation in CRC, illustrating how STING-mediated modulation of the TME synergizes with chemotherapy, radiotherapy, immune checkpoint blockade, and IDO inhibition to improve the effectiveness of combinatorial immunotherapy in CRC.

The cGAS-STING signaling pathway intersects with the DNA response to damage in CRC. Tumors with dMMR/MSI mutations are naturally harnessing STING for immunogenicity, which partly explains their better response to immunotherapy [124]. In non-MSI-high tumors, artificially engaging STING through DNA-damaging treatments or DNA repair inhibitors can convert them into a more inflamed state. The challenge is to maximize immunostimulatory outcomes (e.g., IFNs and T-cell recruitment) while mitigating any immunosuppressive feedback (e.g., PD-L1 induction).

### Safety and delivery issues of STING agonists

STING pathway activation holds strong immunostimulatory promise, yet its clinical translation is hampered by delivery and safety hurdles—particularly in solid tumors such as CRC, where myeloid-rich, immunosuppressive niches can dampen responses [142]. Canonical CDN STING agonists are polyanionic and hydrophilic, which limits membrane permeation and drives rapid clearance from the bloodstream [143, 144]. In early clinical studies, investigators often resorted to intratumoral dosing (e.g., ADU-S100 injections) to achieve meaningful local immune activation, a pattern that highlights how poorly systemic routes reach deep or otherwise inaccessible lesions [145]. At the same time, systemic administration of potent STING agonists can provoke severe inflammation and cytokine release syndrome, reflecting the ubiquitous expression of STING across normal tissues [146]. Too much or poorly targeted STING activation can cause counter-regulation. As one example, STING-driven interferon programs raise PD-L1 levels on tumor cells—pushing T cells toward exhaustion and, in turn, weakening the body’s ability to fight tumors [147]. These challenges highlight the need for more selective delivery approaches that confine STING activation to the tumor milieu.

To overcome these obstacles, recent research has focused on innovative delivery strategies and STING agonist formulations designed to enhance tumor selectivity while reducing systemic toxicity. Another promising route harnesses tumor-resident immunosuppressive cells as targeted delivery hubs for STING agonists. For example, a novel STING agonist prodrug (GB2) was engineered to accumulate in triggering receptor expressed on myeloid cells 2 (TREM2)-expressing macrophages. Administered intravenously in mouse CRC models, GB2 shifted TAMs into a pro-inflammatory phenotype and drove comprehensive tumor regression, without observable systemic toxicity [127, 148]. Taken together, these examples show that improving the pharmacokinetics and tumor specificity of STING agonists can enhance their therapeutic index. Focusing immune activation on the tumor—together with its draining lymph nodes—while sparing normal tissues is the central aim of advanced delivery strategies, which seek to fully realize the anti-tumor potential of STING agonists in CRC. When paired with complementary therapies such as checkpoint blockade, these approaches work to maximize tumor-directed immunostimulation (“igniting” an immune attack) without provoking harmful systemic inflammation [127, 149].

## Delivery systems and nanotechnology

Conventional STING agonists have poor pharmacokinetics. They are cleared from the body quickly, and they can also cause systemic inflammation. Because of this, most trials use intratumoral dosing. The CRC TME is also very suppressive. It needs delivery directly to antigen-presenting cells, including dendritic cells and macrophages, to activate T lymphocytes properly. Nanoparticle carriers can help address these problems. These drugs can last longer in the body and send more of them to the tumor and nearby lymph nodes than to healthy organs. When CDNs or other agonists are placed inside nanoparticles, the drug can reach tumors better and cause fewer adverse consequences in other regions of the body. It also helps the drug enter antigen-presenting cells more easily.

### Rationale for nanoparticle delivery

Using nanocarriers to directly target STING modulators to tumors is one of the most exciting advancements in cGAS-STING-based therapy. The poor pharmacokinetics of conventional STING agonists include off-target inflammation and fast systemic clearance. By extending systematic circulation and improving tumor targeting through the enhanced permeability and retention (EPR) effect [150] and enabling cell-specific targeting, nanoparticle-based delivery systems help overcome these constraints. Encapsulation of STING agonists enables controlled release inside tumor tissues or specific immune subsets [151, 152] and prevents their breakdown. A further enhancement in selectivity is the functionalization of nanocarriers with targeting ligands (e.g., antibodies or carbohydrates). For example, a mannose-coated liposome encapsulating cGAMP greatly enhanced absorption by dendritic cells via mannose receptor-mediated endocytosis, resulting in increased IFN-β synthesis and improved T-cell priming [153]. By improving antigen presentation and adaptive immune activation, DC-targeted cGAMP liposomes potently slowed tumor growth in melanoma models [152]. Originally shown in melanoma, this strategy is easy to translate into CRC by means of antigen delivery to tumor-infiltrating antigen-presenting cells.

### Polymeric and lipid nanocarriers

A range of synthetic nanocarriers have been engineered to deliver STING agonists with improved stability and intracellular retention. Made from PC7A, polymeric micelles are pH-responsive carriers that allow endosomal escape by forming stable complexes with cGAMP [154]. PC7A-cGAMP complexes induce persistent activation of STING and strong expression of IFN-β and CXCL10 in murine MC38 colon carcinoma models, resulting in better tumor control than free cGAMP [155]. Fascinatingly, PC7A nanoparticles also have intrinsic STING-stimulating effects because they cause endosomal stress, underscoring the dual use of such carriers as immune adjuvants and delivery vehicles.

### STING-agonist nanovaccines

Combining STING activation with antigen delivery offers a rational strategy for cancer vaccination. Nanoparticles coencapsulating tumor antigens and STING agonists ensure synchronized delivery to the same antigen-presenting cell, thereby enhancing cross-presentation and T-cell priming. Su et al. [156] developed an acid-responsive polymeric nanovaccine containing both cGAMP and the model antigen ovalbumin (OVA), achieving efficient corelease and immune activation in APCs. In a mouse model of MC38 CRC and other tumor models, this formulation elicited potent CD8^+^ T-cell responses and improved tumor control [157]. Such nanovaccine platforms hold potential for personalization by incorporating patient-specific neoantigens, enabling tailored immunization against individual tumor mutations.

### Metal-based STING platforms

**Fig. 6:**
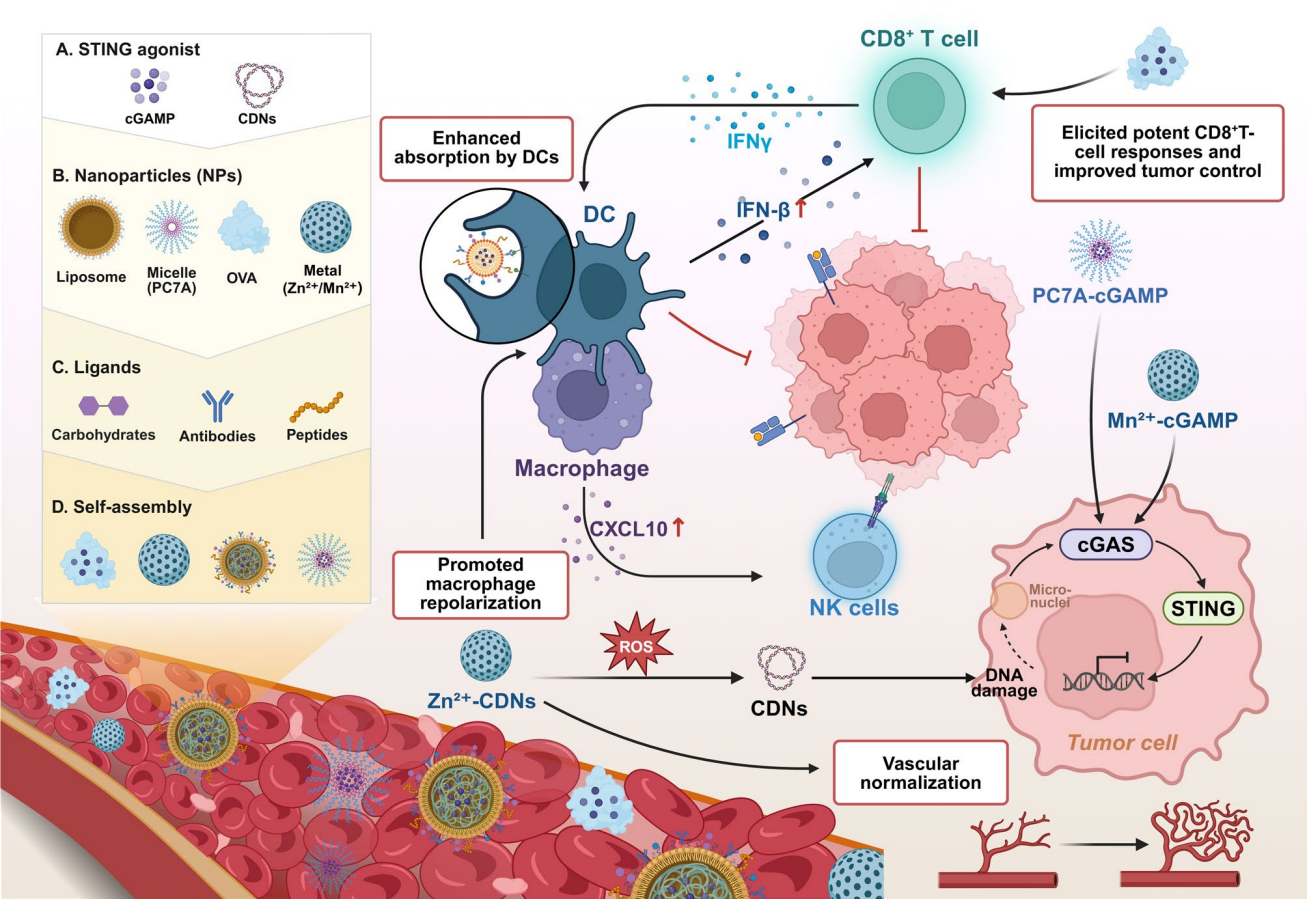
Nanocarrier-mediated STING activation strategies in CRC. Left: (**A**) STING agonists (cGAMP, CDNs); (**B**) nanocarriers (liposomes, PC7A micelles, OVA-loaded NPs, Zn²⁺/Mn²⁺ NPs); (**C**) surface ligands (carbohydrates, antibodies, peptides); (**D**) self-assembled nanoplatforms. Main: Enhanced DC uptake via mannose-functionalized NPs increases the expression of IFN-β, IFN-γ and CXCL10; increases CD8⁺ T-cell and NK cell activation; and induces macrophage repolarization and vessel normalization. Tumor cGAS-STING activation by DNA damage or micronuclei is amplified by Mn²⁺-cGAMP; ROS-responsive Zn²⁺-CDNs promote immune activation and vascular remodeling

Inorganic nanomaterials provide versatile platforms for enhancing STING pathway activation via targeted delivery and cofactor modulation. Manganese-based nanoparticles (Mn²⁺-cGAMPs) increase cGAS activity, increasing cytokine release and antitumor immunity in vivo [158]. Zinc-based polyphosphate nanoparticles coloaded with CDNs target the tumor vasculature and TAMs; Zn^2+^ stabilizes CDNs and directs STING signaling to the TME, promoting vessel normalization and macrophage repolarization [159]. To combine immunotherapy with chemotherapy, robot operating system (ROS)-responsive hybrid nanoparticles have been engineered to codeliver camptothecin and cisplatin. These release drugs in response to tumor-associated ROS, inducing DNA damage and cGAS-STING activation, thereby enhancing dendritic cell maturation and CD8⁺ T-cell infiltration in CRC models [160]. Figure 6 illustrates the diverse nanoparticle platforms and targeting ligands used to enhance STING agonist delivery and immune activation, depicting how these nanocarriers interact with immune cells and elicit potent antitumor responses in CRC.

In the future, moving nanocarrier–STING therapies for CRC will depend on patient selection and good combination strategies. Using biomarkers to guide delivery, such as choosing tumors with high cGAS/STING levels or DNA-sensing signals, may help focus treatment on patients who are more likely to respond. Using smart drug combinations, such as administering STING-loaded nanoparticles together with immune checkpoint inhibitors or ferroptosis inducers, may work better. Ferroptosis is known to change the tumor environment in CRC and may help immune checkpoint inhibitors work more effectively. Important design goals should include ensuring that the drug can escape from endosomes and reach antigen-presenting cells so that it can activate STING inside the cell. It is also important to include personalized neoantigens or other immune-related molecules in the same nanoparticle. In the clinic, the main goals are to improve how well the treatment reaches tumors, reduce side effects, and improve how long the drug stays in the body. As shown in Table 1, these nanomaterials have recently been used in immunotherapy for CRC models. They are mainly used to control size, drug release speed, and functional features, which help guide immune responses more accurately and lower the risk of toxicity.

**Table 1:** Nanomaterials for the delivery of STING agonists in colorectal cancer

Nanomaterial	STING Agonist	CRC Model	Targeting/delivery strategy	Reference
PC7A polymeric nanoparticle (D-SAM, pH-responsive)	cGAMP	MC38 (murine CRC, in vivo)	Endosomal pH-activated polymeric NP for sustained cGAMP release and dual STING stimulation	[169]
Platinum(IV)-silica nanozyme (DMPtNPS)	cGAMP	CT26 (murine) and HCT116 (human) (in vitro & in vivo)	Hypoxia-alleviating Pt-doped silica nanozyme catalyzing ROS to enhance cGAMP STING activation alongside radiotherapy	[170]
Supramolecular HA-CD polymeric NP (HCCSM)	MSA-2 (non-CDN STING agonist)	CT26 (murine, in situ immunotherapy)	CD44-targeted host-guest cyclodextrin-hyaluronic acid NP codelivering MSA-2 and a CPT prodrug for in situ vaccination	[171]
Gold blackbody NP (AuPB)@PDA with Mn²⁺	Mn²⁺ (STING-stimulating ion)	CT26 (orthotopic murine CRC model)	NIR-II photothermal nanoagonist releasing Mn²⁺ to prime cGAS-STING in TAMs and ablate tumor tissue	[172]
ZIF-8 MOF NP in degradable hydrogel (CSZ@Gel)	c-di-GMP (CDG)	CT26 (murine, postresection in situ)	Acid-responsive ZIF-8 NP + in situ hydrogel codelivering CDG and siXkr8; promotes pyroptosis and STING signaling	[173]
Polymeric prodrug nanoparticle (GB2)	MSA-2 (STING agonist prodrug)	MC38 (murine)	TREM2-targeted self-assembling prodrug NP releases MSA-2 in TAMs to reprogram macrophages	[142]
Zn²⁺-coordinated CDN nanoparticle (CDN-Mn²⁺ NP)	c-di-AMP (cyclic dinucleotide)	CT26 (murine, metastatic models)	Metal-organic coordination NP delivering cyclic dinucleotide with Mn²⁺ to amplify STING signaling	[158]

## Clinical translation and future directions

Numerous studies have highlighted the important function of the cGAS‒STING pathway in modulating immunological responses in CRC. The activation of STING signaling, mainly through IFN-I induction, is correlated with improved prognosis and a more immunogenic TME in CRC patients [91, 152]. Although preclinical evidence supports the use of STING agonists as promising immunotherapeutics, several translational hurdles remain. One major challenge is the potential for unwanted side effects caused by systemic STING activation.

### Targeted vs. non-targeted STING activation in nanoplatforms

Exogenous STING agonists are promising for cancer immunotherapy, yet moving them into the clinic remains difficult because of delivery and safety hurdles [161]. Given systemically, these agents can distribute beyond the tumor and activate STING in healthy tissues, provoking severe inflammatory toxicities-cytokine-storm-like reactions with symptoms such as fever and hypotension [162]. This off-target activation argues for tumor-restricted delivery to broaden the therapeutic window [161]. In response, researchers are exploring biomaterial-based delivery systems (nanomedicines) to concentrate STING agonists within tumors and curb systemic exposure [161]. By refining pharmacokinetics and localization, these carriers aim to sustain robust antitumor immune stimulation while reducing adverse effects.

Nanomedicine strategies for STING agonists fall into non-targeted and targeted designs. Non-targeted carriers (polymeric nanoparticles, liposomes, micelles, hydrogels) encapsulate agonists without active ligands, relying on EPR-mediated tumor accumulation or local administration; encapsulation prolongs half-life and enables controlled release but, upon systemic dosing, may still distribute to off-target organs (e.g., liver, spleen), causing unintended STING activation, high circulating cytokines, and autoimmune-like effects, which cap the tolerated dose and can trigger immunosuppressive feedback in the TME (e.g., PD-L1/IDO1 upregulation) [161, 162]. Targeted approaches actively direct payloads to tumor or immune subsets to enhance on-target activation while sparing normal tissues [161, 162]. Antibody–drug conjugates (ADCs) deliver STING agonists to antigen-expressing tumor cells and, via Fcγ-receptor engagement, to tumor-resident dendritic cells/macrophages, yielding preferential intratumoral release and reduced normal-tissue exposure; a HER2-directed STING-ADC exemplified potent intratumoral STING activation with markedly lower systemic cytokines versus an equivalent dose of free agonist [163]. By confining stimulation to the TME, targeted nanomedicines lower off-target inflammatory toxicity while maximizing local antitumor immunity; other ligands (tumor-homing peptides, nucleic-acid aptamers) likewise confer selectivity, and early data indicate that active targeting improves the therapeutic index and may avoid STING-induced T-cell suppression by preventing unnecessary lymphocyte activation [161–163].

Nanomaterial delivery offers a viable route to clinical translation of STING agonists by overcoming key safety and efficacy barriers; judicious use of targeted versus non-targeted designs can maximize tumor-selective activation while limiting systemic inflammation, especially when combined with checkpoint blockade and optimized dosing/scheduling in CRC.

### Tumor-intrinsic STING deficiency

A subset of CRCs lacks cGAS or STING expression [35, 36], limiting direct antitumor action. In such cases, STING agonists may act indirectly through host immune cells. Approaches to address this issue include targeting dendritic cells with nanoparticle-delivered agonists, gene therapy to restore STING expression in tumors, or upstream interventions such as poly ADP‒ribose polymerase (PARP) inhibitors or radiation to promote cytosolic DNA accumulation and engage in residual signaling. The timing and context of STING modulation are crucial. In colitis and long-term intestinal injury, STING inhibition might help prevent CRC progression, but activation at later stages might improve antitumor immunity. A stepwise method, such as the use of STING inhibitors, which are being assessed in several inflammatory diseases, might be effective for treating colitis-associated CRC. The premise of this method is that one must first suppress inflammation and then arm the immunity.

### Translational barriers in nanotechnology

Despite good preclinical data, nanomedicine faces translational barriers: manufacturing scale, reproducibility, regulation, and human PK prediction. It is important to show therapeutic benefit in large animal models. More attention to clinically relevant nanoparticle platforms, instead of many offerings, will facilitate progress. Researchers are investigating ways to create “smart” drugs that can precisely activate immune cells found within the body. Future treatments for CRC can involve a combination of STING agonists along with other modulators of the innate immune system (e.g., retinoic acid-inducible gene I (RIG-I); Toll-like receptors), which can result in the development of a synergistic effect [119]. The use of temporal sequencing also looks promising for activating STING before checkpoint inhibition and then providing continuous antigenic stimulation through chemotherapy (or radiotherapy) [164].

### AI-driven therapeutic

Artificial intelligence (AI)-driven innovations in STING modulator design: Recent advances in AI have begun to address the long-standing limitations of direct STING agonists, which suffer from poor bioavailability—often necessitating intratumoral injection—and dose-limiting systemic inflammatory toxicities, including T-apoptosis [165]. To overcome these challenges, AI-driven approaches are being used to develop indirect STING modulators, particularly those that target Ectonucleotide pyrophosphatase/phosphodiesterase 1 (ENPP1), a cGAMP-degrading ectoenzyme identified as a safer and more effective intervention point than STING itself [166].

For example, Insilico Medicine employed its generative AI platform (Chemistry42) to design ISM5939, a highly selective and orally bioavailable ENPP1 inhibitor. ISM5939 stabilizes extracellular cGAMP within the TME without inducing the systemic cytokine storm or T-cell depletion commonly associated with direct STING agonists [165, 167]. The molecule was discovered within months via an AI-guided, structure-based design pipeline integrating molecular docking, free energy calculations, and in silico ADMET predictions to optimize potency and pharmacokinetic properties [168].

Furthermore, the emergence of next-generation protein structure prediction tools, such as AlphaFold 3, has enabled highly accurate modeling of target–ligand interactions, further enhancing the precision of STING modulator design. Collectively, these AI-driven innovations represent a paradigm shift in STING-targeted drug development, offering a promising path toward safer, more effective immunotherapies.

Finally, validation in clinical trials will be the true test. Initial trials of STING agonists have demonstrated immune pharmacodynamic effects but not yet breakthrough efficacy. As our understanding of dosage, scheduling, and combinations improves, we anticipate more significant clinical responses. Encouragingly, some STING agonists are already in phase II trials for other cancers; lessons from those trials will inform CRC applications.

## Conclusion

The cGAS-STING pathway influences CRC by either inhibiting or facilitating tumor growth. It acts as a tumor suppressor that senses genomic instability and triggers IFN-I responses for the mobilization of DTHC, CD8⁺ T cells and NK cells, especially in MSI-H CRC with high endogenous DNA damage levels. On the other hand, prolonged STING activation in IBD or colitis-associated cancer promotes tumors by inducing chronic inflammation. These 2 adverse effects emphasize the importance of the timing of STING signaling. From a therapeutic point of view, the cGAS‒STING pathway represents an interesting target for next-generation immunotherapy.

Nonetheless, their efficacy on their own is limited, requiring combination strategies and targeted delivery. Systems based on nanoparticles are particularly promising, as they permit localized STING activation and the codelivery of complementary therapeutics to enhance the antitumor response while limiting systemic toxicity. The impact of cGAS–STING activity is mediated by the disease setting, as highlighted by findings that collectively underscore its importance as a core immune surveillance mechanism in CRC. Modulating this pathway in a strategic way can help improve immunotherapy results by activating it in tumors and potentiating it in inflamed settings.

## Data Availability

No datasets were generated or analysed during the current study.

## Abbreviations

5-FU: 5-fluorouracil
ADCs: Antibody–drug conjugates
ATR: Ataxia telangiectasia and Rad3-related protein
Batf3: Basic leucine zipper ATF-like transcription factor 3
BCL-2: B-cell lymphoma 2
CAFs: Cancer-associated fibroblasts
CAR: Chimeric antigen receptor
CCL5: C-C motif chemokine ligand 5
CDNs: Cyclic dinucleotides
cGAMP: Cyclic GMP-AMP
cGAS-STING: Cyclic GMP-AMP synthase–stimulator of interferon genes
CRC: Colorectal cancer
CTLs: Cytotoxic T lymphocytes
CXCL10: C-X-C motif chemokine ligand 10
CXCL9: C-X-C motif chemokine ligand 9
DAMPs: Damage-associated molecular patterns
DCs: Dendritic cells
Dhx9: DExH-box helicase 9
dMMR: Deficient mismatch repair
DMXAA: Vadimezan
dsDNA: Double-stranded DNA
DSS: Dextran sulfate sodium
EPR effect: Enhanced permeability and retention effect
ER: Endoplasmic reticulum
FOXP3: Forkhead box protein P3
GATA3: GATA-binding protein 3
GPX4: Glutathione peroxidase 4
GSH: Glutathione
IBD: Inflammatory bowel disease
ICI: Immune checkpoint inhibitor
IDO: Indoleamine 2,3-dioxygenase
IECs: Intestinal epithelial cells
IFN-β: Interferon-beta
IFN-I: Type I interferons
IKK: IκB kinase
IL-1β: Interleukin-1 beta
IL-18: Interleukin-18
IL-6: Interleukin-6
IRF3: Interferon regulatory factor 3
ISGs: Interferon-stimulated genes
LRRC8C: Leucine-rich repeat-containing 8 VRAC subunit C
MDSCs: Myeloid-derived suppressor cells
MHC: Major histocompatibility complex
MSI-H: Microsatellite instability-high
NCOA4: Nuclear receptor coactivator 4
NF-κB: Nuclear factor kappa-B
NK cells: Natural killer cells
NLRP3: NOD-,LRR- and pyrin domain-containing protein 3
NRF2: Nuclear factor erythroid 2–related factor 2
OMVs: Outer membrane vesicles
p53: Tumor protein p53
PARP1: Poly [ADP‒ribose] polymerase 1
PD-1: Programmed cell death protein 1
PD-L1: Programmed cell death ligand 1
RIG-I: Retinoic acid-inducible gene I
SHP1: Src homology region 2 domain-containing phosphatase-1
SLC7A11: Solute carrier family 7 member 11
SOCS1: Suppressor of cytokine signaling 1
STAT3: Signal transducer and activator of transcription 3
Syk: Spleen tyrosine kinase
TAMs: Tumor-associated macrophages
TBK1: TANK-binding kinase 1
Th2 cell: T helper 2 cell
TME: Tumor microenvironment
TNF-α: Tumor necrosis factor-alpha
TNFRSF9: Tumor necrosis factor receptor superfamily member 9
Tregs: Regulatory T cells
TREM2: Triggering receptor expressed on myeloid cells 2
VDAC1: voltage-dependent anion channel protein 1
